# Sedation-related complications during ERCP in liver transplant recipients: a retrospective cohort study

**DOI:** 10.1186/s12876-026-05098-5

**Published:** 2026-07-06

**Authors:** Nihal Gökbulut Özaslan, Mehmet Şahap, Gokhan Erdem, Bülent Ödemiş

**Affiliations:** 1 Department of Anesthesiology and Reanimation, Ankara Bilkent City Hospital, Ankara, Turkey; 2 Department of Gastroenterology, Ankara Bilkent City Hospital, Ankara, Turkey

**Keywords:** ERCP, Liver transplantation, Anesthesia, Propofol, Sedation

## Abstract

**Background:**

Liver transplant recipients frequently undergo endoscopic retrograde cholangiopancreatography (ERCP) for biliary complications and may be at increased risk of sedation-related adverse events. However, data regarding sedation-related outcomes in this population remain limited. This study aimed to evaluate the incidence, characteristics, and potential predictors of sedation-related complications during ERCP in liver transplant recipients.

**Methods:**

We conducted a retrospective cohort study including adult liver transplant recipients who underwent ERCP under anesthesiologist-administered deep sedation between July 2021 and June 2022. For patients who underwent multiple ERCP procedures, only the first procedure was included. Sedation-related complications were defined as the occurrence of one or more of the following events during ERCP or within 60 min after the procedure: oxygen desaturation, hypotension, bradycardia, apnea, or airway intervention. Univariable and multivariable logistic regression analyses were performed to identify factors associated with complications.

**Results:**

A total of 76 patients were included. Sedation-related complications occurred in 21 procedures (27.6%). Oxygen desaturation was the most common event (22.4%), followed by hypotension (9.2%), apnea (3.9%), and bradycardia (2.6%). Airway intervention, consisting exclusively of jaw thrust maneuvers, was required in 10 procedures (13.2%). No patient required bag-mask ventilation, supraglottic airway placement, endotracheal intubation, or procedure termination due to sedation-related instability. In univariable analyses, longer procedure duration and higher propofol dose were associated with complications. No independent predictors were identified after multivariable adjustment. However, given the limited sample size and number of events, the study may have been underpowered to detect modest associations.

**Conclusions:**

Sedation-related cardiopulmonary events were common but predominantly mild and transient. No independent predictors were identified after multivariable adjustment; however, the study may have been underpowered to detect modest associations.

**Trial registration:**

Not applicable.

**Supplementary Information:**

The online version contains supplementary material available at 10.1186/s12876-026-05098-5.

## Background

Orthotopic liver transplantation is the standard treatment for end-stage liver disease and has been shown to substantially improve both survival and quality of life [1]. However, despite these advances, post-transplant complications remain common, particularly biliary complications such as anastomotic strictures and leaks, which have been reported in 5–34% of recipients [2]. These complications may lead to significant clinical consequences, including cholestasis, cholangitis, graft dysfunction, and even graft loss [3].

Endoscopic retrograde cholangiopancreatography (ERCP) is currently considered the first-line modality for both the diagnosis and treatment of biliary complications following liver transplantation, with high technical and clinical success rates reported in the literature [4]. Given the complexity and discomfort associated with ERCP, adequate sedation or anesthesia is essential to ensure procedural success and patient safety.

Sedation-related cardiopulmonary events, particularly hypoxemia and hypotension, are among the most frequently reported complications during ERCP in the general population [5]. Although these events are usually transient and manageable, they may still require immediate intervention and therefore remain clinically relevant during procedural sedation [6].

Liver transplant recipients may represent a particularly vulnerable group due to multiple comorbidities, ongoing immunosuppressive therapy, and altered physiological reserve [2].

Despite the widespread use of ERCP in this population, data specifically evaluating sedation-related complications and their associated risk factors in liver transplant recipients remain limited and heterogeneous [7]. A deeper understanding of these risks is essential for optimizing sedation strategies and improving procedural safety.

The aim of this study was to retrospectively evaluate the incidence and characteristics of sedation-related complications in liver transplant recipients undergoing ERCP at our center and to identify potential risk factors associated with these events.

## Methods

This study was designed and reported according to the STROBE statement for observational studies.

### Study design and setting

This retrospective cohort study was conducted at a tertiary referral center. The study included liver transplant recipients who underwent ERCP under sedation between July 2021 and June 2022. Ethical approval (E1-22-2712, 29/06/2022) was obtained from the local ethics committee before data collection. The requirement for informed consent was waived by the ethics committee due to the retrospective nature of the study.

As this was a retrospective cohort study, clinical trial registration was not required.

### Study population

Adult liver transplant recipients who underwent ERCP under sedation were eligible for inclusion. If a patient underwent more than one ERCP procedure during the study period, only the first ERCP procedure was included in the analysis to avoid repeated observations from the same individual.

Eligible patients were adults (≥ 18 years) with a history of liver transplantation who underwent ERCP under sedation. Patients younger than 18 years, pregnant women, procedures performed under general anesthesia, and cases with incomplete records were excluded.

No eligible cases were excluded because of incomplete anesthetic or procedural records during the study period.

### ERCP procedure

The indication for ERCP was post-transplant biliary complications, including biliary anastomotic stricture and leak.

During this study period, all ERCP procedures were performed using anesthesiologist-administered sedation. All ERCP procedures were performed by an experienced, high-volume endoscopist.

### Sedation protocol

Sedation was administered and monitored by the anesthesiology team. Standard monitoring, including continuous electrocardiography, pulse oximetry, and noninvasive blood pressure monitoring, was applied throughout the procedure. Propofol was used as the primary sedative agent according to the institutional sedation protocol (0.5 mg/kg bolus followed by 100–150 µg/kg/min infusion) in all procedures. All patients received continuous oxygen supplementation (4 L/min via nasal cannula), and all procedures were performed under continuous capnographic monitoring.

When necessary, adjunct sedative agents including midazolam (1 mg) and fentanyl (50 µg) were administered at the discretion of the anesthesiologist. Sedation depth was titrated to achieve a Ramsay Sedation Score (RSS) of 5–6, consistent with deep sedation, to ensure optimal procedural conditions. Total propofol dose (mg), midazolam use (yes/no), and opioid use (yes/no) were recorded.

### Data collection

Data were retrospectively collected from hospital electronic medical records, endoscopy reports, and anesthesia/sedation charts. The following variables were recorded for each ERCP procedure: Demographic and clinical variables, including age, sex, American Society of Anesthesiologists (ASA) physical status classification, and comorbid conditions including diabetes mellitus (DM), hypertension, coronary artery disease (CAD), and chronic obstructive pulmonary disease (COPD), were recorded. In addition, the Model for End-Stage Liver Disease (MELD) score at the time of ERCP was recorded.

Transplant-related variables included the time interval between liver transplantation and ERCP (years). Procedure-related variables included procedure duration (minutes), defined as the time from endoscope insertion to its withdrawal, and sedative medication use. The study period (July 2021 to June 2022) was selected based on the availability of complete electronic anesthesia records and consistent capnographic monitoring at our institution from that date onward; extension beyond 12 months was not feasible within the timeframe of this retrospective analysis.

All patients were receiving maintenance immunosuppressive therapy at the time of ERCP. However, detailed information regarding specific immunosuppressive agents (e.g., tacrolimus, cyclosporine, or mycophenolate mofetil), drug dosages, serum drug levels, and detailed biochemical parameters (ALT, AST, ALP, GGT, bilirubin, INR, and albumin) was not consistently available in the retrospective medical records and therefore could not be reliably included in the analysis.

### Outcome measures

The primary outcome was the occurrence of a sedation-related complication during ERCP or within the recovery period (defined as up to 60 min after completion of the procedure).

Sedation-related complications were defined as the occurrence of one or more of the following events: desaturation (peripheral oxygen saturation < 90% for at least 10 s), hypotension (systolic blood pressure < 90 mmHg or a decrease of ≥ 20% from baseline), bradycardia (heart rate < 40 beats per minute), apnea (cessation of respiratory effort for ≥ 20 s or any respiratory pause requiring intervention), or airway intervention (including jaw thrust, airway adjunct placement, or endotracheal intubation). These definitions were adapted from the available institutional anesthesia records and are consistent with the framework used in ERCP-sedation research [8].

Each adverse event was recorded separately. For the primary analysis, a composite binary outcome variable (“sedation-related complication,” yes/no) was created and defined as the occurrence of one or more predefined adverse events during a single ERCP procedure. Patients experiencing multiple adverse events during the same procedure were counted once in the composite outcome analysis. The threshold for desaturation (SpO2 < 90%) used in this study differs slightly from the hypoxemia definition proposed in the 2020 ESGE guideline on ERCP-related adverse events (SpO2 < 85%), which may limit direct comparability with studies using ESGE-standardized definitions [8].

Because individual adverse events occurred infrequently, a composite endpoint was used to facilitate evaluation of overall sedation-related complications within the study cohort.

### Statistical analysis

Statistical analysis was performed using IBM SPSS Statistics version 29.0. Continuous variables were first assessed for normality using the Shapiro-Wilk test. Variables with approximately normal distributions were expressed as mean ± standard deviation; non-normally distributed variables were expressed as median (interquartile range). Categorical variables were presented as numbers and percentages.

For comparisons between ERCP procedures with and without sedation-related complications, continuous variables were analyzed using the Student’s t-test or Mann–Whitney U test, as appropriate. Categorical variables were compared using the chi-square test or Fisher’s exact test.

To identify factors associated with sedation-related complications, univariable analyses were first performed. Variables considered clinically relevant and those showing significance in univariable analysis were entered into a multivariable logistic regression model. Given the sample size and anticipated number of events, the number of variables included in the final model was limited to avoid overfitting. Candidate predictors included age, ASA physical status, MELD score, procedure duration, total propofol dose, and time since transplantation. A two-sided p value of < 0.05 was considered statistically significant.

To avoid violation of the independence assumption inherent in standard logistic regression, only the first ERCP procedure per patient was included in the analysis; therefore, no additional adjustment for within-patient clustering was required. Given that only 21 events were available for analysis, the events-per-variable (EPV) ratio in the multivariable model was approximately 3.5, falling below the commonly recommended threshold of 10. Accordingly, the number of candidate predictors was limited to six clinically relevant variables to minimize the risk of overfitting. Results of the multivariable model should therefore be interpreted with caution, as estimates may be unstable and modest associations may remain undetected.

Potential collinearity between procedure duration and total propofol dose was acknowledged, as longer procedures inherently require greater cumulative sedative exposure. Although formal collinearity diagnostics (e.g., variance inflation factors) were not performed, both variables were retained in the model given their independent clinical relevance. Their interdependence represents a potential source of residual confounding and should be considered when interpreting the multivariable results.

## Results

**Fig. 1:**
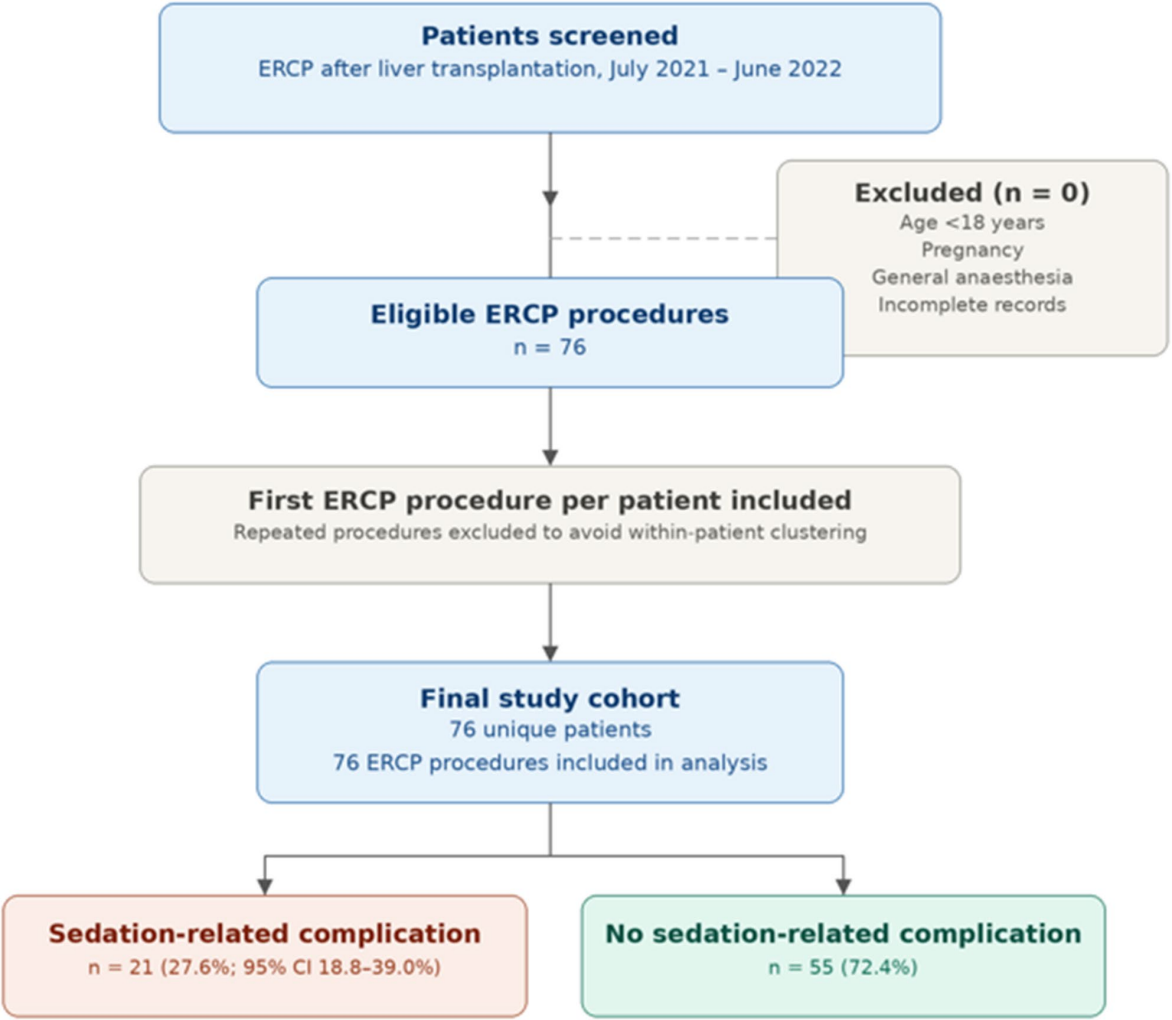
STROBE participant flow chart

A total of 76 liver transplant recipients were included in the study. For patients who underwent more than one ERCP procedure during the study period, only the first ERCP procedure was included in the analysis. Therefore, the final study cohort consisted of 76 unique patients and 76 ERCP procedures. No eligible patients were excluded because of incomplete records (Fig. 1).

The demographic and clinical characteristics of the study population are summarized in Table 1. The mean age was 50.2 ± 9.7 years, and the mean interval between liver transplantation and ERCP was 5.9 ± 2.9 years. Fifty patients (65.8%) were male and 26 (34.2%) were female. According to the ASA classification, 35 patients (46.1%) were classified as ASA II and 41 (53.9%) as ASA III. Diabetes mellitus, hypertension, coronary artery disease, and chronic obstructive pulmonary disease were present in 22 (28.9%), 35 (46.1%), 12 (15.8%), and 9 patients (11.8%), respectively.

**Table 1:** Baseline characteristics of the study population

Variable		*n*
Age (years)		50.2 ± 9.7
Male sex		50 (65.8%)
ASA score	II	35 (46.1%)
	III	41 (53.9%)
Diabetes mellitus		22 (28.9%)
Hypertension		35 (46.1%)
Coronary artery disease		12 (15.8%)
COPD		9 (11.8%)
MELD score		9.4 ± 1.7
Time from transplantation to ERCP (years)		5.9 ± 2.9

Values are expressed as mean ± SD or n (%)

*COPD* Chronic obstructive pulmonary disease, *MELD *Model for End-Stage Liver Disease, *ASA* American Society of Anesthesiologists

Procedural and sedation-related characteristics are presented in Table 2. The indication for ERCP in all patients was post-transplant biliary anastomotic leak or stricture.

**Table 2:** Procedural and sedation characteristics

Variable	*n*
ERCP indication - post-transplant biliary complications	100%
Procedure duration (min)	19.5 ± 9.7
Propofol dose (mg)	173.2 ± 65.1
Midazolam use	51 (67.1%)
Fentanyl use	8 (10.5%)

Values are expressed as mean ± SD or n (%)

The mean ERCP procedure duration was 19.5 ± 9.7 min. The mean total propofol dose administered during ERCP was 173.2 ± 65.1 mg.

Adjunct sedative agents were used in some procedures. Midazolam (1 mg) was administered in 51 procedures (67.1%), whereas 25 procedures (32.9%) were performed without midazolam. Fentanyl (50 µg) was administered in 8 procedures (10.5%).

Sedation-related complications observed during ERCP are summarized in Table 3. Overall, 21 of 76 procedures (27.6%; 95% CI 18.8–39.0%) were associated with at least one sedation-related complication. Some procedures were associated with more than one adverse event; however, each procedure was counted only once in the composite outcome analysis.

**Table 3:** Sedation-related complications

Complication	*n* (%)
Desaturation	17 (22.4%)
Hypotension	7 (9.2%)
Bradycardia	2 (2.6%)
Apnea	3 (3.9%)
Airway intervention (jaw thrust)	10 (13.2%)

Values are expressed as number (percentage)

The most frequently observed complication was oxygen desaturation, which occurred in 17 procedures (22.4%). Hypotension was observed in 7 procedures (9.2%), whereas bradycardia and apnea occurred in 2 (2.6%) and 3 (3.9%) procedures, respectively. Airway intervention, consisting exclusively of jaw thrust maneuvers, was required in 10 procedures (13.2%).

No patient required bag-mask ventilation, oral or nasal airway placement, supraglottic airway device insertion, or endotracheal intubation. All respiratory events were transient and were successfully managed with supportive interventions. No procedure was terminated prematurely because of sedation-related instability, and no patient required advanced airway management.

Comparison of clinical and procedural variables between procedures with and without sedation-related complications is presented in Table 4. Age, MELD score, ASA physical status, and time since transplantation were comparable between groups (all *p* > 0.05).

**Table 4:** Univariable analysis of factors associated with sedation-related complications

Variable	Complication + (*n* = 21)	Complication – (*n* = 55)	Unadjusted OR	95% CI	*p* value
Age (years)	48.7 ± 10.8	50.4 ± 10.3	0.98	0.94–1.03	0.52
ASA III	11 (52.4%)	30 (54.5%)	0.42	0.15–1.19	0.103
Procedure duration (min)	22 ± 10	16 ± 8	1.09	1.03–1.15	0.01
Propofol dose (mg)	190 ± 60	150 ± 60	1.01	1.007–1.025	0.007
MELD score	9.2 ± 2	9.7 ± 2	0.96	0.71–1.3	0.3
Time from transplantation to ERCP (years)	5.9 ± 3.3	6.0 ± 2.9	0.96	0.8–1.14	0.93

Values are presented as mean ± SD or n (%). Continuous variables were compared using Student’s t-test or Mann–Whitney U test, as appropriate, and categorical variables using chi-square or Fisher’s exact test

In univariable analyses, both procedure duration (OR 1.09, 95% CI 1.03–1.15; *p* = 0.01) and total propofol dose (OR 1.01, 95% CI 1.00–1.02; *p* = 0.007) were significantly associated with sedation-related complications.

Variables considered clinically relevant, including age, ASA physical status, MELD score, time since transplantation, procedure duration, and total propofol dose, were subsequently entered into a multivariable logistic regression model (Table 5). However, neither procedure duration nor total propofol dose remained statistically significant after adjustment, and no independent predictors of sedation-related complications were identified. Given the limited sample size and number of events, the multivariable analysis may have been underpowered to detect modest associations.

**Table 5:** Multivariable logistic regression analysis of factors associated with sedation-related complications

Variable	OR	95% CI	*p* value
Age	0.98	0.93–1.03	0.392
ASA III	0.80	0.26–2.43	0.693
MELD score	1.00	0.73–1.36	0.992
Time since transplantation	0.91	0.74–1.11	0.348
Procedure duration	1.02	0.92–1.13	0.715
Propofol dose	1.01	0.99–1.03	0.203

Adjusted for age, ASA physical status, MELD score, time since transplantation, procedure duration, and total propofol dose

## Discussion

The present study evaluated sedation-related outcomes in liver transplant recipients undergoing ERCP for post-transplant biliary complications. Sedation-related cardiopulmonary events occurred in approximately one-quarter of procedures. Desaturation was the most common adverse event, whereas severe complications requiring advanced airway management, procedure termination, or intensive care admission were not observed.

Biliary complications are among the most common problems following liver transplantation, and ERCP has become the primary therapeutic approach for their management [9]. As ERCP procedures may be uncomfortable and technically demanding, adequate sedation is essential to ensure optimal procedural conditions. Propofol is widely preferred for ERCP sedation due to its rapid onset and favorable recovery profile [10]. However, sedation-related cardiopulmonary events remain a concern, particularly in patients with significant comorbidities.

The observed rate of sedation-related complications is broadly consistent with previously published ERCP sedation studies, in which respiratory and hemodynamic events represent the most commonly reported adverse events. Similar to prior reports, most events in our cohort were transient and were successfully managed with supportive measures without escalation to advanced airway interventions [11, 12]. Of note, a recent prospective study reported a desaturation rate of 22.6% among 232 ERCP procedures performed under nurse-administered propofol sedation, closely comparable to the 22.4% rate observed in our cohort [13]. Furthermore, a 2025 systematic review and meta-analysis encompassing over 967,000 procedural sedations confirmed that advanced endoscopic procedures such as ERCP are associated with substantially higher rates of hypoxia compared with diagnostic endoscopy [14].

In our cohort, sedation-related events were predominantly mild and mainly consisted of transient respiratory changes, particularly oxygen desaturation. This finding is consistent with previous studies conducted in the general ERCP population, where hypoxemia and hypotension are the most commonly reported complications [11, 15].

Importantly, all observed respiratory events were transient and successfully managed with simple supportive interventions. Airway interventions were limited to basic maneuvers such as jaw thrust, and no patient required advanced airway management.

In univariable analyses, both longer procedure duration and higher propofol dose were associated with sedation-related complications. However, neither association remained statistically significant after adjustment in the multivariable model. These findings suggest that the observed relationships may have been influenced by confounding factors such as procedure duration, procedural complexity, or limited statistical power. Therefore, the present findings do not identify propofol dose as an independent predictor of sedation-related complications. Rather, the observed association in univariable analyses likely reflects procedural factors that accompany greater sedative requirements [12]. It should also be noted that a substantial proportion of procedures involved the co-administration of adjunct agents (midazolam in 67.1% and fentanyl in 10.5% of cases), which may have contributed to sedation depth and respiratory effects in ways that are not fully captured by propofol dose alone. This polypharmacy context limits the reliability of propofol dose as an isolated predictive factor and represents an additional source of residual confounding.

In contrast, patient-related factors such as age, ASA physical status, and MELD score were not significantly associated with sedation-related complications. Although ASA classification is frequently used as a marker of peri-procedural risk, no significant association was identified in the present cohort. Given that only 21 events occurred, the study had limited statistical power for multivariable modeling. Therefore, failure to identify independent predictors should be interpreted cautiously and may reflect insufficient power rather than absence of true associations.

Despite these concerns, the absence of severe cardiopulmonary events in the present cohort is reassuring. However, given the retrospective design, limited sample size, and lack of a control group, the findings should not be interpreted as definitive evidence of safety.

Given that most observed events were respiratory in nature, careful monitoring and prompt supportive interventions remain important during ERCP sedation.

Data specifically evaluating sedation-related outcomes during ERCP in liver transplant recipients remain limited. Therefore, the primary contribution of the present study is not to identify a novel independent predictor of sedation-related complications, but rather to characterize the frequency and nature of these complications in liver transplant recipients undergoing ERCP. Although the association between greater sedative exposure and sedation-related complications is not unexpected, the existing literature specifically evaluating this patient population remains sparse.

### Limitations of the study

Several limitations should be acknowledged. First, this was a retrospective single-center study, which may limit the generalizability of the findings and introduce selection bias. Second, the study lacked a non-transplant control group and formal post-procedural follow-up, limiting comparisons with the general ERCP population and preventing assessment of delayed complications. Third, the relatively small sample size and limited number of events may have reduced statistical power and contributed to possible overfitting of the multivariable model.

Fourth, detailed information regarding immunosuppressive regimens was not consistently available. Therefore, the potential influence of specific immunosuppressive agents, drug dosages, serum levels, or drug–drug interactions with sedative medications could not be evaluated. As immunosuppressive therapy may affect drug metabolism and physiological responses, residual confounding related to these factors cannot be excluded. Fifth, detailed respiratory monitoring variables (such as minimum oxygen saturation, duration of desaturation episodes, and capnography-derived parameters) were not consistently available. Similarly, detailed ERCP complexity characteristics (including sphincterotomy, balloon dilation, stent exchange, or leak therapy) were not systematically recorded in the anesthesia charts reviewed for this study and therefore could not be incorporated into the analysis. Sixth, several potentially relevant patient-level variables—including body mass index (BMI), inpatient versus outpatient status, and the presence of cholangitis or sepsis at the time of ERCP—were not consistently documented and could not be reliably extracted from the retrospective records; their potential influence on sedation-related outcomes therefore cannot be excluded. Seventh, the MELD score was used as a measure of disease burden at the time of ERCP; however, this score was originally developed to predict mortality in patients with cirrhosis and may not fully reflect overall comorbidity burden in post-transplant recipients with restored graft function. A broader measure such as the Charlson Comorbidity Index may have been more appropriate, but could not be reliably calculated from the available retrospective records.

To avoid clustering effects associated with repeated procedures in the same patient, only the first ERCP procedure performed during the study period was included. In addition, all ERCP procedures were performed for post-transplant biliary leak or stricture, which may limit the applicability of the findings to other ERCP indications in transplant recipients. Finally, the use of a composite endpoint allowed evaluation of sedation-related events despite the relatively low frequency of individual complications. However, because the composite outcome included both transient physiological disturbances and more clinically relevant events, the reported complication rate may overestimate the incidence of clinically significant sedation-related morbidity. Future studies with larger cohorts may permit separate analysis of individual adverse events according to their clinical relevance.

## Conclusion

In this cohort of liver transplant recipients undergoing ERCP, sedation-related cardiopulmonary events occurred in approximately one-quarter of procedures and were predominantly mild and transient. All events were successfully managed without procedure termination, advanced airway intervention, or intensive care admission.

Although procedure duration and propofol dose were associated with complications in univariable analyses, no independent predictors were identified after adjustment. Given the absence of a non-transplant control group and the limited sample size, further prospective studies are needed to better define risk factors and compare outcomes with those of the general ERCP population.

## Supplementary Information

Supplementary Material 1.

## Data Availability

The datasets used and analyzed during the current study are available from the corresponding author on reasonable request.

## Abbreviations

ERCP: Endoscopic retrograde cholangiopancreatography
RSS: Ramsay Sedation Score
DM: Diabetes mellitus
CAD: Coronary artery disease
COPD: Chronic obstructive pulmonary disease
MELD: Model for End-Stage Liver Disease
ASA: American Society of Anesthesiologists
