# Supplementary material for: Sedation-related complications during ERCP in liver transplant recipients: a retrospective cohort study
Source: BMC Gastroenterol. 2026 Jul 6;26:499. doi: 10.1186/s12876-026-05098-5 (PMC13463787; doi:10.1186/s12876-026-05098-5)
Supplement: Supplementary file 1 [file 12876_2026_5098_MOESM1_ESM.doc]

STROBE Statement—Checklist of items that should be included in reports of ***cohort studies***

|  | Item No | Recommendation |
| --- | --- | --- |
| **Title and abstract** | 1 | (*a*) Indicate the study’s design with a commonly used term in the title or the abstract: Retrospective cohort study |
|  |  | (*b*) Provide in the abstract an informative and balanced summary of what was done and what was found: Structured abstract describing background, methods, results and conclusions. |
| Introduction | | |
| Background/rationale | 2 | Explain the scientific background and rationale for the investigation being reported: The limited evidence regarding sedation-related outcomes during ERCP in liver transplant recipients and the need to characterize complication rates in this population. |
| Objectives | 3 | State specific objectives, including any prespecified hypotheses: To evaluate the incidence, characteristics, and predictors of sedation-related complications during ERCP in liver transplant recipients. |
| Methods | | |
| Study design | 4 | Present key elements of study design early in the paper: Retrospective single-center cohort study. |
| Setting | 5 | Describe the setting, locations, and relevant dates, including periods of recruitment, exposure, follow-up, and data collection: Tertiary referral liver transplant center; July 2021–June 2022. |
| Participants | 6 | (*a*) Give the eligibility criteria, and the sources and methods of selection of participants. Describe methods of follow-up: Adult liver transplant recipients undergoing ERCP under anesthesiologist-administered deep sedation. Only the first ERCP procedure per patient was included. |
|  |  | (*b*) For matched studies, give matching criteria and number of exposed and unexposed: Not applicable. |
| Variables | 7 | Clearly define all outcomes, exposures, predictors, potential confounders, and effect modifiers. Give diagnostic criteria, if applicable: Age, sex, ASA status, MELD score, comorbidities, time since transplantation, procedure duration, propofol dose, sedative adjuncts, and sedation-related complications. |
| Data sources/ measurement | 8* | For each variable of interest, give sources of data and details of methods of assessment (measurement). Describe comparability of assessment methods if there is more than one group: Data were collected retrospectively from electronic medical records, anesthesia charts, and endoscopy reports. |
| Bias | 9 | Describe any efforts to address potential sources of bias: To reduce clustering bias, only the first ERCP procedure for each patient was included. Study limitations related to retrospective design were acknowledged. |
| Study size | 10 | Explain how the study size was arrived at: All eligible patients during the study period were included. |
| Quantitative variables | 11 | Explain how quantitative variables were handled in the analyses. If applicable, describe which groupings were chosen and why: Continuous variables were expressed as mean ± SD and analyzed as continuous variables. |
| Statistical methods | 12 | (*a*) Describe all statistical methods, including those used to control for confounding: Student’s t-test, chi-square test, univariable and multivariable logistic regression analyses were performed. |
|  |  | (*b*) Describe any methods used to examine subgroups and interactions: Not applicable. |
|  |  | (*c*) Explain how missing data were addressed: Cases with incomplete anesthetic or procedural records were excluded. |
|  |  | (*d*) If applicable, explain how loss to follow-up was addressed: Not applicable due to retrospective design. |
|  |  | (*e*) Describe any sensitivity analyses: Not performed. |
| Results | | |
| Participants | 13* | (a) Report numbers of individuals at each stage of study—eg numbers potentially eligible, examined for eligibility, confirmed eligible, included in the study, completing follow-up, and analysed: Final cohort included 76 unique liver transplant recipients undergoing ERCP. |
|  |  | (b) Give reasons for non-participation at each stage: Exclusions shown in STROBE flow diagram. (in figüre 1) |
|  |  | (c) Consider use of a flow diagram: Figure 1 |
| Descriptive data | 14* | (a) Give characteristics of study participants (eg demographic, clinical, social) and information on exposures and potential confounders: Baseline demographic and clinical characteristics summarized in Table 1. |
|  |  | (b) Indicate number of participants with missing data for each variable of interest: Detailed biochemical and immunosuppressive data were not consistently available. |
|  |  | (c) Summarise follow-up time (eg, average and total amount): Not applicable. |
| Outcome data | 15* | Report numbers of outcome events or summary measures over time: Sedation-related complications summarized in Table 3. |
| Main results | 16 | (*a*) Give unadjusted estimates and, if applicable, confounder-adjusted estimates and their precision (eg, 95% confidence interval). Make clear which confounders were adjusted for and why they were included: Univariable and multivariable logistic regression analyses presented in Tables 4 and 5. |
|  |  | (*b*) Report category boundaries when continuous variables were categorized: Not applicable. |
|  |  | (*c*) If relevant, consider translating estimates of relative risk into absolute risk for a meaningful time period: Not applicable. |
| Other analyses | 17 | Report other analyses done—eg analyses of subgroups and interactions, and sensitivity analyses: Multivariable logistic regression analysis performed. |
| Discussion | | |
| Key results | 18 | Summarise key results with reference to study objectives: Sedation-related complications occurred in 27.6% of procedures and were predominantly mild and transient. |
| Limitations | 19 | Discuss limitations of the study, taking into account sources of potential bias or imprecision. Discuss both direction and magnitude of any potential bias: Retrospective design, single-center setting, limited sample size, absence of control group, and incomplete availability of some clinical variables. |
| Interpretation | 20 | Give a cautious overall interpretation of results considering objectives, limitations, multiplicity of analyses, results from similar studies, and other relevant evidence: Findings interpreted in the context of previous ERCP sedation literature and study limitations. |
| Generalisability | 21 | Discuss the generalisability (external validity) of the study results: Findings may not be generalizable to other ERCP indications or institutions. |
| Other information | | |
| Funding | 22 | Give the source of funding and the role of the funders for the present study and, if applicable, for the original study on which the present article is based: No external funding was received.(page:declarations) |

*Give information separately for exposed and unexposed groups.

**Note:** An Explanation and Elaboration article discusses each checklist item and gives methodological background and published examples of transparent reporting. The STROBE checklist is best used in conjunction with this article (freely available on the Web sites of PLoS Medicine at http://www.plosmedicine.org/, Annals of Internal Medicine at http://www.annals.org/, and Epidemiology at http://www.epidem.com/). Information on the STROBE Initiative is available at http://www.strobe-statement.org.
